# Surgical resection or bevacizumab for treatment of radiation necrosis-associated cerebral edema in patients with brain metastases: A single center experience

**DOI:** 10.1093/noajnl/vdag120

**Published:** 2026-05-11

**Authors:** Miles E Dijkstra†, Joost W Schouten†, Lotte Plegt, Anna L Peulen, Damon van Hees, Cleo Slagter, Bibi L J Bouwen, Martin J van den Bent, Joost L M Jongen, Eelke M Bos

**Affiliations:** Department of Neurosurgery, Brain Tumor Center, Erasmus MC Cancer Institute, Erasmus University Medical Center, Rotterdam, The Netherlands; Department of Neurosurgery, Brain Tumor Center, Erasmus MC Cancer Institute, Erasmus University Medical Center, Rotterdam, The Netherlands; Department of Neurosurgery, Brain Tumor Center, Erasmus MC Cancer Institute, Erasmus University Medical Center, Rotterdam, The Netherlands; Department of Neurosurgery, Brain Tumor Center, Erasmus MC Cancer Institute, Erasmus University Medical Center, Rotterdam, The Netherlands; Department of Neurosurgery, Brain Tumor Center, Erasmus MC Cancer Institute, Erasmus University Medical Center, Rotterdam, The Netherlands; Department of Radiotherapy, Brain Tumor Center, Erasmus MC Cancer Institute, Erasmus University Medical Center, Rotterdam, The Netherlands; Department of Neurosurgery, Brain Tumor Center, Erasmus MC Cancer Institute, Erasmus University Medical Center, Rotterdam, The Netherlands; Department of Neuro-Oncology, Brain Tumor Center, Erasmus MC Cancer Institute, Erasmus University Medical Center, Rotterdam, The Netherlands; Department of Neuro-Oncology, Brain Tumor Center, Erasmus MC Cancer Institute, Erasmus University Medical Center, Rotterdam, The Netherlands; Department of Neurosurgery, Brain Tumor Center, Erasmus MC Cancer Institute, Erasmus University Medical Center, Rotterdam, The Netherlands

**Keywords:** bevacizumab, brain metastasis, neurosurgery, radiation necrosis, steroids

## Abstract

**Introduction:**

Cerebral radiation necrosis (RN) is a frequent adverse event of stereotactic radiotherapy (SRT) for brain metastases. This retrospective, single-center cohort study compares surgical resection with bevacizumab for the management of RN-associated cerebral edema in patients with brain metastases following SRT.

**Methods:**

Data were extracted from the electronic health records of patients >18 years old that were operated or received bevacizumab for metastatic cerebral RN at Erasmus MC. Inclusion criteria were histopathologically confirmed or a clinical and radiological diagnosis of cerebral RN. Primary outcomes included dexamethasone usage, post-treatment edema volume, and adverse event rates.

**Results:**

Among 62 patients treated with resection and 32 with bevacizumab, both interventions resulted in significant reductions in cerebral edema and dexamethasone usage. Surgical resection resulted in a higher rate of dexamethasone discontinuation (81.0% vs 46.7%, *P* < .0001) and a lower incidence of grade 2 or higher adverse events (3.2% vs 43.8%) than bevacizumab. Overall survival did not differ between groups.

**Conclusions:**

Surgical resection can serve as a principal therapeutic strategy for RN-associated cerebral edema in appropriately selected cases where procedural risk is deemed acceptable. Prospective studies are warranted to refine criteria for patient selection and to optimize management strategies for RN occurring in the context of brain metastases.

Key Points

- Resection and bevacizumab both achieved significant reductions in edema and dexamethasone usage.
- Surgical resection resulted in a higher rate of complete dexamethasone discontinuation.
- Less grade 2 or higher adverse events occurred in the surgery arm of this study.

Importance of the Study

This study addresses a clinical dilemma by directly comparing 2 effective strategies, surgical resection and bevacizumab, for managing radionecrosis (RN)-associated cerebral edema. Both treatments rapidly reduced edema and steroid dependence, but our data show surgical intervention achieved more frequent complete steroid discontinuation and provided immediate mass-effect relief. These advantages are especially relevant for patients eligible for immunotherapy, where minimizing corticosteroid exposure is critical for the effectivity of the treatment. The retrospective design and careful surgical selection introduce bias by indication, so safety and efficacy comparisons are not definitive; nevertheless, the findings highlight that, when patients are well selected within a multidisciplinary team, resection merits consideration as a first-line option. Given rising brain metastasis incidence and increase in cases of RN that follows, these results inform treatment choice and future prospective evaluation.

Brain metastases (BM) are the most common intracranial tumors in adults, representing about 50% of intracranial neoplasms, occurring in up to 20% of patients with systemic cancer.^1^ BM represent a major source of morbidity and mortality in oncology care.^1^ Advances in systemic therapies in the last 10-20 years have improved survival in many patients, making local control of BM an increasingly important therapeutic goal. Radiotherapy, both whole-brain radiotherapy (WBRT) and stereotactic radiotherapy (SRT), are a cornerstones of treatment, offering effective tumor control and symptom palliation.^2^^,^^3^ In recent years, there has been a significant shift away from the use of WBRT and towards SRT. However, one of the most challenging delayed adverse events of SRT for BM is cerebral radionecrosis (RN), a form of treatment-induced brain injury characterized by irreversible parenchymal necrosis, blood-brain barrier disruption, and inflammatory edema.^4^

RN typically presents around 6-12 months after treatment and may be radiographically indistinguishable from tumor progression, complicating clinical decision-making.^5^^,^^6^ Symptomatic RN occurs in approximately 5%-25% of SRT-treated patients, depending on factors such as total radiation dose, number of fractions, irradiated volume, re-stereotactic irradiation and prior therapies.^7^ The standard initial management for RN involves corticosteroids, which reduce perilesional edema and alleviate mass effect, though its long-term use is limited by systemic toxicity.^8^ In addition, corticosteroids may render medical treatment with immune cell checkpoint inhibitors less effective.^9^ Bevacizumab, a monoclonal antibody against vascular endothelial growth factor (VEGF), has emerged as an option with growing evidence for efficacy in reducing both steroid dependence and imaging abnormalities.^10^ In patients with refractory symptoms, diagnostic uncertainty or large necrotic lesions causing mass effect, surgical resection may be preferable over bevacizumab.^11^ Surgery has been shown to be an effective treatment option for patients suffering from RN, achieving significant edema reduction and reducing steroid dependency.^12^ Understanding the comparative effectiveness and appropriate selection criteria for these treatment modalities is critical for optimal care of patients with BM experiencing RN.

A recent systematic review pooled outcomes of bevacizumab and surgery for radionecrosis, reporting symptom stability or improvement in 86% (95% CI: 77%-92%) for bevacizumab and in 89% (95% CI: 81-96%) for surgery.^13^ Bevacizumab and surgery offer distinct benefits and drawbacks. Bevacizumab, though less invasive, was associated with medical adverse events in 24.4% of patients.^13^ Surgery, while more invasive, had a low incidence of severe adverse events including neurologic complications and oncologic treatment delays, in 0%-2% of the patients.^13^ A further comparison of these options will help tailor treatment to individual patient needs.

This single center cohort study aimed to investigate the efficacy of bevacizumab and surgical resection for RN, assessed through dexamethasone usage and post-treatment edema volume. Additionally, adverse event rates for both treatments were compared using CTCAE grade, a clinically validated and widely applied classification system. This study represents a relatively large cohort, strengthening the robustness of our analyses.

## Methods

### Patients

Data were extracted from the electronic health records of patients >18 years old that were operated (from 2010 to 2022) or received bevacizumab (from 2019 to 2023) for metastatic cerebral RN at Erasmus MC. Inclusion criteria were histopathologically confirmed in case of surgery or a clinical and radiological diagnosis of cerebral RN in case of treatment with bevacizumab after SRT. If no follow-up data were available at both the 3-month and 6-month intervals, patients were excluded. Since overall survival and dexamethasone use were outcome measures, patients that necessitated secondary treatment, either resection or bevacizumab, were excluded from these analyses.

A histopathological diagnosis of RN was established using the conclusions of the pathological report. A clinical and radiological diagnosis of cerebral RN was based on consensus in the multidisciplinary brain tumor board, if no biopsy was available.

### Patient Characteristics

The following data were collected from all patients: date of birth, date of death, follow-up time, date of progression, gender, date of presentation with BM, date of start of RN treatment (either date of resection or start of bevacizumab treatment), maximum diameter of the largest brain metastasis, depth of the metastasis, number of metastases, extent of resection of necrosis, primary tumor, surgical and adverse events following treatment, presence of epilepsy, use of anti-epileptic drugs (AED) and presence of leptomeningeal metastases. Follow-up time was defined as date of treatment of to date of either death or censoring. Presence of leptomeningeal metastases was based on clinical or radiologic diagnosis by a neuro-oncologist and/or radiologist. The largest diameter was measured on the coronal view of the pre-treatment scan as the distance between the outer cortex of the tumor. The depth of the metastases was defined as the minimum distance between the outer margin of the enhancing tumor on contrast-enhanced T1-weighted MRI scans and the cerebral cortex. Since most patients were asymptomatic while under steroids when additional treatment for radiation necrosis (RN) was initiated, the main endpoints for this study were dexamethasone dosage/discontinuation and edema control and were analyzed as proxies for clinical symptoms. Surgical and medical adverse events were classified using the common terminology criteria for adverse events (CTCAE). Epilepsy data were collected for 6 months pre- and post-treatment.

### Surgical Treatment

Patient selected for surgery underwent craniotomy under general anesthesia, using standard neurosurgical adjuncts such as neuronavigation, intraoperative ultrasound, ultrasonic aspiration at the neurosurgeon’s discretion. Patients underwent MRI within 48 hours of surgery.

### Bevacizumab Treatment

Bevacizumab was administered intravenously at a dose of 7.5 mg/kg every 3 weeks, for a maximal number of 4 doses. The MRI on which a diagnosis of RN was established was used as baseline, a follow-up MRI was made 12 weeks after start of bevacizumab treatment and every 3 months thereafter.

### Analysis of Dexamethasone Usage

For patients who received multiple resections or multiple series of bevacizumab treatments, only the dexamethasone usage pre- and post-treatment of the first intervention was collected. In our center, patients are regularly given a short course of high dose dexamethasone (3-5 days) perioperatively. This high dose was not interpreted as the pre-intervention dose. The average daily dose of dexamethasone given in mg/day within 3 weeks of the intervention was used as the pre-intervention dose for all patients. The daily dose of dexamethasone was collected before treatment and at 2, 6,12, and 24 weeks after treatment. A mixed effects analysis was used to compare quantitative variables within and between the groups, supplemented with *Šidák’s* multiple comparisons test.

### Analysis of Edema Volume

Edema was quantitatively assessed on FLAIR or T2 weighted MR images. For both groups, if a secondary therapy (either bevacizumab or craniotomy) was administered later, only the edema measurements taken before the initiation of the secondary therapy were included in the primary analysis.

Edema was assessed using volumetric measurement in Brainlab Elements Smartbrush 4.0.0.108.^14^ Volumes were measured at multiple time points: baseline (the last scan made pre-treatment), within 48 hours post-treatment, and at 3, 6, 9, and 12 months after treatment (when available). These measurements were normalized to a baseline value of 100. Both the raw and normalized edema measurements were plotted with the median and interquartile range (IQR) used to illustrate the data distribution.

Patients that received treatment for recurrence of edema were separately assessed. The results of these analyses are stated in the supplemental results.

### Statistical Analysis

Statistical analyses were performed using GraphPad Prism 10.1.0 software. In this exploratory analysis, a *P* < .05 was used to determine a significant difference. We used Kaplan-Meier analyses supplemented with the Log-rank test to determine the overall survival between the groups. Fisher’s exact test was used to compare categorical variables. The independent *t*-test was used to compare means and Mann-Whitney *U* test to compare medians.

## Results

### Patient Characteristics

In the surgical records, 529 patients operated for brain metastasis were identified, of which 361 had received prior radiotherapy. We found 72 patients who initially underwent craniotomy for (histopathologically confirmed) RN. A total of 34 patients with BM received bevacizumab as their initial treatment for suspicion of RN after SRT.

In the craniotomy group, 2 patients were excluded due to an initial edema volume that was below 1 cm^3^, and 8 were excluded due to insufficient follow-up data. In the bevacizumab group, 2 patients were excluded for missing follow-up data.

The final dexamethasone usage analysis included 53 patients in the craniotomy group and 30 in the bevacizumab group. The final edema analysis included 62 and 32, respectively. Mean time from first SRT to RN treatment was 16.8 months. Mean follow-up time in this cohort was 51.5 months in the bevacizumab group, and 58.1 months for the surgical group.

As shown in Table 1, there was no significant difference in diameter of the largest metastasis. The median being 29.5 mm (IQR: 23.9-36.0) in the resection group, and 26.0 mm (IQR: 21.3-32.5) in the bevacizumab group. The depth of the metastases was significantly larger in the bevacizumab group, with a median of 0.0 mm (IQR: 0.0-7.7) and a mean of 4.03 mm (SD: 6.84) compared to a median 0.0 mm (IQR: 0.0-0.0) and mean 1.41 mm (SD: 3.23) in the surgical group. Patients in the bevacizumab group were affected significantly more often by multiple metastases (46.9%) compared to the surgery group (24.2%). Lung carcinoma was the most common primary tumor in both groups, followed by melanoma. Epilepsy was remarkably prevalent in both groups: in 59.7% of the surgical group and in 75.0% in the bevacizumab group.

**Table 1.** Patient characteristics per treatment group

	Resection (*n* = 62) *n* (%)	Bevacizumab (*n* = 32) *n* (%)	*P* value
Sex			.515
* *Male	26 (41.9)	16 (50.0)	
* *Female	36 (58.1)	16 (50.0)	
Age			
* *Mean ± SD	58.7 ± 10.7	62.0 ± 10.4	.153
* *Median [IQR]	59.5 [52.0-67.0]	63.5 [57.5-69.8]	.065
Diameter largest metastasis (mm)			
* *Mean ± SD	32.1 ± 10.7	27.9 ± 8.7	.059
* *Median [IQR]	29.5 [23.9-36.0]	26.0 [21.3-32.5]	.123
Depth of metastasis (mm)			
* *Mean ± SD	1.4 ± 3.2	4.0 ± 6.8	**.047**
* *Median [IQR]	0.0 [0.0-0.0]	0.0 [0.0-7.7]	**.034**
Multiple metastases	15 (24.2)	15 (46.9)	**.030**
Edema volume (cm^3^)			
* *Mean ± SD	113.5 ± 72.4	96.0 ± 51.4	.617
* *Median [IQR]	108.7 [46.5-169.8]	87.9 [59.3-144.8]	.455
Location of primary tumor			.154
* *Lung	32 (51.6)	22 (68.8)	
* *Melanoma	14 (22.6)	4 (12.5)	
* *Mamma	10 (16.1)	1 (3.1)	
* *Other	5 (8.1)	4 (12.5)	
* *Unknown	1 (1.6)	1 (1.6)	
Leptomeningeal disease	3 (4.8)	1 (3.1%)	1.000
Pre-treatment epilepsy	18 (29.0)	13 (40.6)	.287
Pre-treatment AED use	30 (48.4)	17 (53.1)	.531

Abbreviation: AED, anti-epileptic drug. Bold values represent statistical significance (<.05).

Leptomeningeal disease at presentation was present in 3 (4.8%) cases in the surgery group, and 1 (3.1%) case for the bevacizumab cohort.

### Survival Analysis

#### Overall survival

No significant difference was found in the Kaplan-Meier analysis of overall survival between the resection group (*n* = 72) and the bevacizumab group (*n* = 32) (*P* = .70, Supplementary Figure S1). The resection group had a median overall survival of 3.31 years, compared to 3.35 years in the bevacizumab group.

### Analyses of Dexamethasone Use

#### Dexamethasone dosage

**Figure 1.**
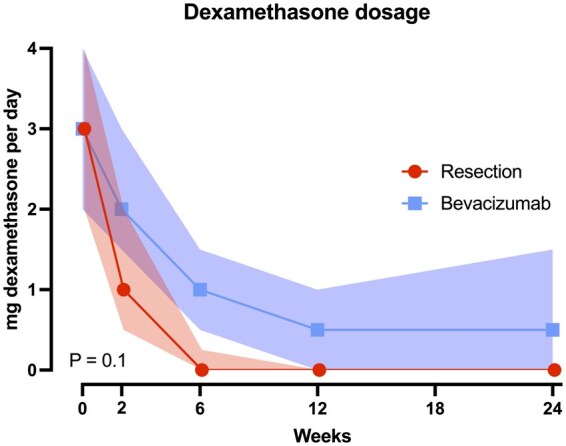
Dexamethasone dosage over time per treatment, median +/− 95% CI.

No significant difference was found in the dosage of dexamethasone use pre- and post-intervention between surgical patients (*n* = 63) and bevacizumab patients (*n* = 32) (*P* = .1). There seems to be a trend in which surgical patients require less dexamethasone usage post-intervention (Figure 1).

#### Dexamethasone discontinuation

**Figure 2.**
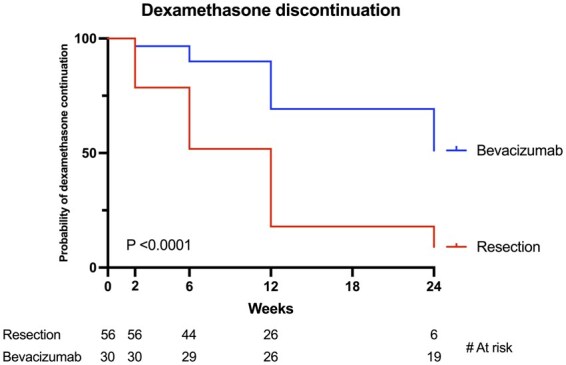
Kaplan-Meier curves for dexamethasone discontinuation per treatment.

A significant difference was found in the discontinuation of dexamethasone discontinuation post-intervention between surgical patients and bevacizumab patients (*P* < .0001) (Figure 2). In total, 47 (81.0%) surgical patients were able to completely discontinue dexamethasone, compared to 14 (46.7%) bevacizumab patients. A hazard ratio of 3.01 (95% CI: 1.83-4.99) was found for stopping dexamethasone post-intervention in the surgical group compared to the bevacizumab group.

#### Adverse events

Adverse events were experienced by 9.0% of surgical patients. There was one patient (1.5%) who had to undergo an additional operation in which an external ventricular drain was placed, due to postsurgical edema in the posterior cranial fossa. Of bevacizumab patients, 62.5% experienced an adverse event. This difference was statistically significant (*P* < .001). Bevacizumab had to be discontinued by 6 patients (19.4%) due to adverse events (Table 2).

**Table 2.** Adverse events per treatment group

Surgical group (*n* = 62)		Bevacizumab group (*n* = 32)		*P* value
CTCAE grade	*N* (%)		*N* (%)	<.001
1	6 (9.7)	1	6 (18.7)	
2	2 (3.2)	2	13 (40.7)	
3	0 (0.0)	3	1 (3.1)	
4	0 (0.0)	4	0 (0.0)	
Adverse event	*N* (%)			
Temporary paresis	3 (3.8%)	Hypertension	5 (15.6%)	
Hyponatremia	2 (3.2%)	Pneumonia	4 (12.5%)	
Cerebral infarction	1 (1.6%)	Pulmonary embolism	3 (9.4%)	
Neurologic decline	1 (1.6%)	Wound stagnation	2 (6.3%)	
Hyperglycemia	1 (1.6%)	Neurologic decline	2 (6.3%)	
		DVT	1 (3.1%)	
		Hyponatremia	1 (3.1%)	
		Dermatitis	1 (3.1%)	
		Colitis	1 (3.1%)	
Total adverse events	8 (12.9%)		20 (62.5%)	
Total patients with adverse events	6 (9.0%)		17 (53.1%)	
One adverse event	4 (6.5%)		14 (43.8%)	
Multiple adverse events	2 (3.2%)		3 (9.4%)	
Reoperations	1 (1.5%)	Premature discontinuation	6 (19.4%)	
		Received 1 dose	2 (6.5%)	
		Received 2 doses	1 (3.2%)	
		Received 3 doses	3 (9.7%)	

Abbreviation: CTCAE, common terminology criteria for adverse events.

### Analysis of Edema Groups

#### Edema volume pre- and post-initial treatment

**Figure 3.**
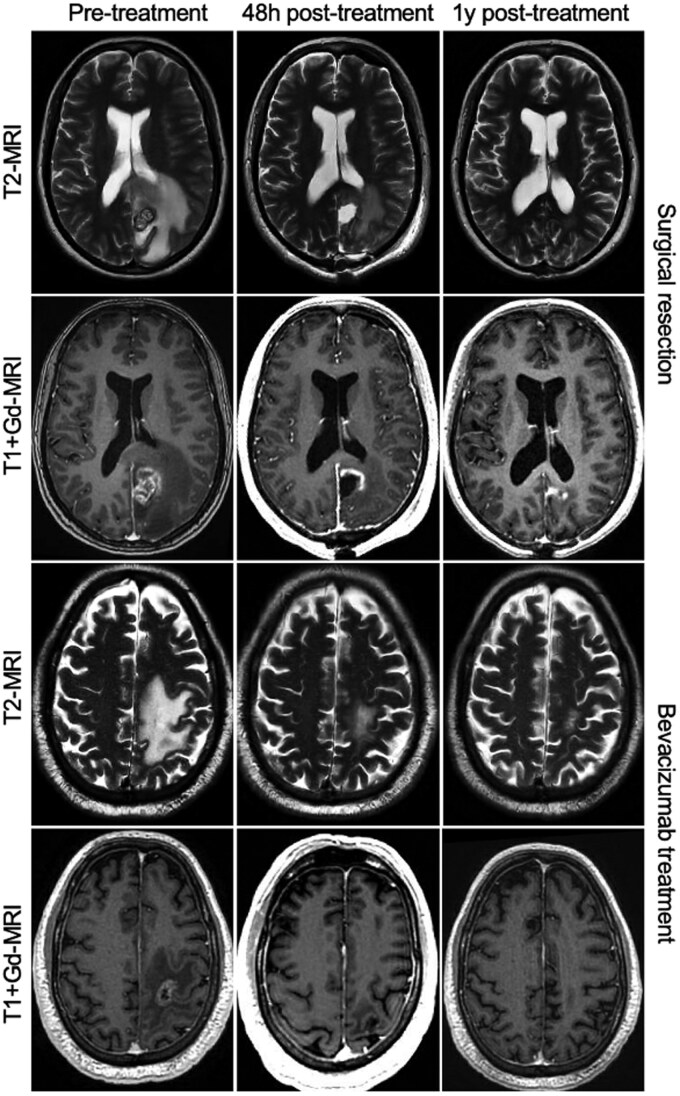
Scans of a selected representative patient of each group, based on median pre-treatment edema volumes.

**Figure 4.**
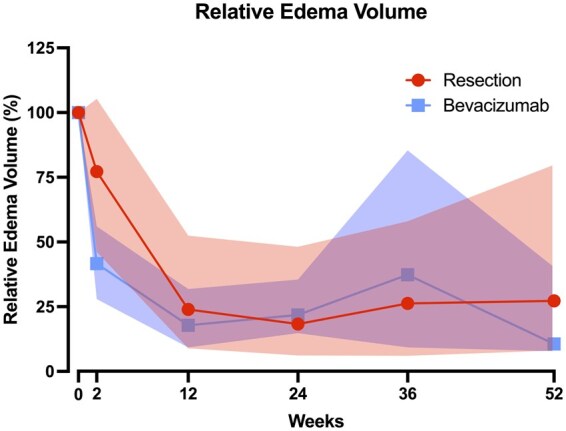
Normalized edema volume in percentage, median + IQR, per treatment group over follow-up duration.

Both treatment groups show a decrease in edema, as seen in the illustrative cases in Figure 3. Median normalized post-treatment volumes being 77.22 (IQR: 46.16-105.29) in the resection group, and 51.37 (IQR: 27.96-56.04). This difference was not significant. After 3 months the decrease continued to 23.11 (IQR: 8.7-52.7) in the surgical group and 18.43 (IQR: 11.5-33.3) in the bevacizumab group (Figure 4).

#### Edema volume pre- and post-secondary treatment

Nine patients in the craniotomy group received bevacizumab as treatment for recurrent edema. The median time between craniotomy and bevacizumab was 11.7 months. In these cases, 5 of the patients required bevacizumab due to edema from a different lesion than the one operated on. In the 4 treated with bevacizumab for the same lesion, edema reduced by more than 70% (Supplementary Figure S2).

Only one patient who received a craniotomy post-bevacizumab was included. This craniotomy was performed 20 months after bevacizumab. This patient, directly post-treatment, showed an edema decrease of 54.1%, which further improved to 89.6% 12 months post-surgery (Supplementary Figure S3).

### Presence of Epilepsy and Use of AED

#### Surgical cohort epilepsy data

In the surgical group, as described in Table 1,^15^ (29.0%) patients had at least one epileptic seizure in the 6 months prior to surgery, and 30 (48.4%) patients used AED. In the 6 months after surgery only 8 (12.9%) patients suffered a seizure, which represents a significant improvement (*P* = .038), whilst AED use remained stable, at 31 patients (50.0%).

#### Bevacizumab cohort epilepsy data

As shown in Table 1, epileptic seizures occurred in 13 (40.6%) patients in the 6 months preceding bevacizumab and 17 (53.1%) used AED. Seizures occurred in 8 (25%) patients in the 6 months after administration (*P* = .23), and 19 (59.4%) patients used AED.

## Discussion

### Summary of Findings and Limitations

Both surgical resection and medical treatment using bevacizumab were effective in treating RN associated edema. Within weeks after either treatment, dexamethasone usage was reduced, and imaging showed a clear reduction in cerebral edema. In an exploratory analysis, epilepsy prevalence was lower in the 6-months after treatment. Both interventions have different safety profiles. Whereas surgical morbidity was low in our study, these cases were selected for surgery in multidisciplinary meetings, likely excluding patients with higher risk of adverse events and neurological deterioration from surgical resection. Patients that were not eligible for surgery were likely continued on steroids or treated with bevacizumab. In addition, as expected due to the retrospective nature of this study, patient characteristics differed on several points between the 2 treatment modalities, indicating the possible presence of bias by indication. Tumor diameter and depth of the tumor from the brain surface were different between the groups: in the surgical resection group tumors were located more superficially and tended to be larger.

All bevacizumab patients in this study were treated with a commonly used regime, as described. Alternative regimens reducing bevacizumab dose may change efficacy while aiming for reduction of side effects. Although this retrospective series does not present a randomized comparison and we cannot conclude with certainty that surgery has a better safety profile compared to bevacizumab due to the differences mentioned above, the data do indicate that in well-selected patients’ surgical morbidity is very low and this treatment needs to be considered with suspected RN as a first-line treatment for management.

### Advantages of Resection as Treatment for RN

Surgical treatment results depend on careful patient selection. Surgery in patients with tumors is immediately effective in reducing mass effect and symptoms of raised intracranial pressure, and complete resection is associated with seizures reduction.^16^ Additionally, if medical treatment with corticosteroids or bevacizumab fails, surgery must be considered. An additional and major advantage of surgery is the histopathological confirmation of the nature of the lesion, which will greatly influence postoperative treatment decisions.

Complete discontinuation of steroids was reached significantly more often after surgery, and the dose was reduced faster. These results might be biased due to different specialties being responsible for dexamethasone tapering. After surgery, the neurosurgeon reduced dexamethasone dose, while in the bevacizumab patients, the neuro-oncologist was responsible for this. Imaging results do not show a quicker reduction in edema volume after surgery compared to bevacizumab. In addition, tumors in the bevacizumab-treated patients were seated more deeply in the brain, which might affect the ability to reduce steroid dose.

### Importance of Reductions in Dexamethasone Use

Minimizing dexamethasone exposure is particularly important in patients with BM who are candidates for immunotherapy. Corticosteroids exert broad immunosuppressive effects that can counteract the mechanism of immune checkpoint inhibitors, effectively blunting their anti-tumor efficacy.^9^^,^^17^ Chronic steroid use is so counterproductive to immunotherapy that nearly half of trials with ICIs exclude patients on sustained high-dose steroids.^9^ Given that modern immunotherapies can substantially prolong survival even in metastatic brain cancer, every effort should be made to avoid unnecessary immunosuppression. Therefore, interventions that control RN-associated edema while sparing or reducing corticosteroid use may not only improve neurological outcomes and avoid debilitating side effects of steroids but also preserve patients’ ability to fully benefit from immunotherapy.^12^^,^^15^^,^^18^

### Implications of the Findings

With the rising incidence of BM and the need for local control in the brain, SRT is more frequently used. The incidence of symptomatic radionecrosis has increased over the past 2 decades, likely driven by more extensive use of SRT, namely (fractionated) SRT for larger metastases and repeated SRS for recurrent BM, as well as the growing use of immune checkpoint inhibitors.^19^ These results are therefore timely and can support clinicians in management of patients who are symptomatic due to presumed RN. We propose that surgery or bevacizumab should be considered for these patients at an earlier stage to prevent the dependence on and side effects of long-term steroid use.

In our experience, the remnants of the previously irradiated metastasis can frequently be recognized on MRI, surrounded by RN. During surgical resection, this irradiated metastasis remnant can frequently be recognized and removed. In these cases, it appeared removing only the irradiated metastasis, and a part of the RN is enough to achieve the beneficial effects on steroid and edema reduction (as illustrated by Figure 3, Supplementary Figures S6 and S7). Currently, we do not strive for complete resection of necrotic material based on MRI. An important additional benefit of surgical resection is the possibility for histopathological analysis of the tissue, confirming the diagnosis of radionecrosis, but also indicating whether there is vital tumor tissue in the sample, which can guide additional treatment choices. As histopathological confirmation is not always feasible, recent advances in neuroradiological imaging, including diffusion-weighted imaging and dynamic susceptibility contrast MRI, have shown promise in non-invasively distinguishing radionecrosis from tumor progression, further underscoring the value of interdisciplinary collaboration in managing these patients.^20^^,^^21^

It is not immediately clear which of these treatments is more cost-effective. The costs of bevacizumab itself are lower compared to the costs of surgery in most healthcare systems. While bevacizumab treatment is performed in daycare, and surgery requires patients to be admitted, the rate of adverse events is higher in bevacizumab-treated patients, likely incurring additional costs. An analysis of all treatment-related costs is necessary to answer whether one of these treatments is more cost-effective.

### Summary

Both bevacizumab and surgical resection reduce steroid dependency quickly and effectively in patients with brain metastasis-associated radionecrosis. Both treatments should be considered in patients who are steroid dependent in a multidisciplinary team with neurosurgical involvement. Our study is limited by bias by indication resulting in baseline differences between the groups. However, surgery resulted in a higher percentage of patients in which steroids could be completely discontinued and in fewer grade 2 or higher adverse events, showing that surgery is a strong option for patients who are steroid dependent due to RN. In our view, when it is deemed safe, surgery is the treatment of first choice for these patients.

## Supplementary Material

vdag120_Supplementary_Data

## Data Availability

Data used for this study will be made available upon reasonable request.
