# Supplementary material for: Surgical resection or bevacizumab for treatment of radiation necrosis-associated cerebral edema in patients with brain metastases: A single center experience
Source: Neurooncol Adv. 2026 May 11;8(1):vdag120. doi: 10.1093/noajnl/vdag120 (PMC13197578; doi:10.1093/noajnl/vdag120)
Supplement: vdag120_Supplementary_Data [file vdag120_supplementary_data.docx]

Supplementary material

Supplementary Results 1.1 Overall survival graph

*Supplementary figure 1: Kaplan-Meier curve of the overall survival per treatment P:0.70*

Supplementary Results 1.2 Edema volumes of patients receiving secondary treatment

*1.2.1 Bevacizumab post resection*

**

*Supplementary Figure 2: Edema volume in the bevacizumab post-craniotomy group*

*1.2.2 Resection post bevacizumab*

**

*Supplementary Figure 3: Edema volume in the resection post-bevacizumab group*

**Supplementary results 1.3 Comparative results of complete and incomplete necrosis resection**

In 17 (27.4%) of the 62 patients in the surgical cohort the radiation necrosis was completely resected. For 43 (69.4%) an incomplete resection was achieved, and for 2 (3.2%) this was unclear.

Dexamethasone discontinuation and edema volumes post-operatively were compared between complete and incomplete necrosis resection. For both these outcomes no difference was found.

**

*Supplementary figure 4: Kaplan-Meier curve of dexamethasone discontinuation between complete and incomplete necrosis resection within the surgical cohort. NS = non-significant*

*
Supplementary figure 5: Comparison between of edema volumes post-complete and incomplete resection of necrosis*
